# Major Superficial White Matter Abnormalities in Huntington's Disease

**DOI:** 10.3389/fnins.2016.00197

**Published:** 2016-05-23

**Authors:** Owen R. Phillips, Shantanu H. Joshi, Ferdinando Squitieri, Cristina Sanchez-Castaneda, Katherine Narr, David W. Shattuck, Carlo Caltagirone, Umberto Sabatini, Margherita Di Paola

**Affiliations:** ^1^Morphology and Morphometry for NeuroImaging Lab, Clinical and Behavioural Neurology Department, IRCCS Fondazione Santa LuciaRome, Italy; ^2^Neuroscience Department, University of Rome “Tor Vergata”Rome, Italy; ^3^Ahmanson Lovelace Brain Mapping Center, Neurology, University of California Los AngelesLos Angeles, CA, USA; ^4^IRCCS Casa Sollievo della SofferenzaSan Giovanni Rotondo, Italy; ^5^CSS-MendelRome, Italy; ^6^Lega Italiana Ricerca Huntington FoundationRome, Italy; ^7^Radiology Department, IRCCS Santa Lucia FoundationRome, Italy; ^8^Department of Psychiatry and Clinical Psychobiology, University of Barcelona, IDIBAPSBarcelona, Spain; ^9^Clinical and Behavioural Neurology Department, IRCCS Fondazione Santa LuciaRome, Italy; ^10^Neuroradiology, University of Magna GraeciaCatanzaro, Italy; ^11^Human Studies Department, Libera Università Maria SS. Assunta (LUMSA)Rome, Italy

**Keywords:** Huntington's disease, white matter, DTI diffusion, intracortical myelin, CAG repeat length, MRI, oligodendrocyte, movement disorders

## Abstract

**Background:** The late myelinating superficial white matter at the juncture of the cortical gray and white matter comprising the intracortical myelin and short-range association fibers has not received attention in Huntington's disease. It is an area of the brain that is late myelinating and is sensitive to both normal aging and neurodegenerative disease effects. Therefore, it may be sensitive to Huntington's disease processes.

**Methods:** Structural MRI data from 25 Pre-symptomatic subjects, 24 Huntington's disease patients and 49 healthy controls was run through a cortical pattern-matching program. The surface corresponding to the white matter directly below the cortical gray matter was then extracted. Individual subject's Diffusion Tensor Imaging (DTI) data was aligned to their structural MRI data. Diffusivity values along the white matter surface were then sampled at each vertex point. DTI measures with high spatial resolution across the superficial white matter surface were then analyzed with the General Linear Model to test for the effects of disease.

**Results:** There was an overall increase in the axial and radial diffusivity across much of the superficial white matter (*p* < 0.001) in Pre-symptomatic subjects compared to controls. In Huntington's disease patients increased diffusivity covered essentially the whole brain (*p* < 0.001). Changes are correlated with genotype (CAG repeat number) and disease burden (*p* < 0.001).

**Conclusions:** This study showed broad abnormalities in superficial white matter even before symptoms are present in Huntington's disease. Since, the superficial white matter has a unique microstructure and function these abnormalities suggest it plays an important role in the disease.

## Introduction

Huntington's disease (HD) is a neurodegenerative autosomal dominant disorder caused by increased CAG repeats leading to an increased accumulation of mutant huntingtin, and formation of intranuclear inclusions and eventually resulting in brain damage. This damage has long been suspected to start in the striatum (Esmaeilzadeh et al., 2011), however, most areas of the brain show abnormalities. Abnormal white matter has been hypothesized to be potentially independently vulnerable in HD (Bartzokis et al., 2007). Recently, a number of studies have shown that in HD and even in pre-symptomatic (Pre-HD) subjects there are abnormalities across much of the large long range white matter fibers (Di Paola et al., 2012, 2014; Phillips et al., 2014, 2015) (here referred to as deep white matter). The novelty of the present study was to focus on the integrity of the short-range association fibers (U-fibers) and intracortical myelin that lay at the interface between the cortical gray matter and white matter (here called the superficial white matter–please see Table 1 for a brief overview). The hypothesis behind this investigation was, that the superficial white matter could be sensitive to disease processes because of its location and its unique characteristics. Indeed, the superficial white matter is the last region to myelinate and this results in high plasticity but also high vulnerability (Bartzokis, 2004). Secondly, the oligodendrocytes in the superficial white matter myelinate many axon segments with fewer wraps than in the deep white matter (Butt and Berry, 2000), which gives them less protection against damage and makes the fibers more sensitive to impairments (Haroutunian et al., 2014). Finally, since the superficial white matter has been shown to be especially vulnerable to the normal aging process (Phillips O. R. et al., 2013), Alzheimer's disease (Phillips et al., 2016), and schizophrenia (Phillips et al., 2011) it may also be vulnerable to disease processes in HD.

**Table 1:** **Basic differences between superficial white matter and deep white matter**.

**Superficial White Matter (SWM)**	**Deep White Matter**
Short fibers	Long fibers
Small diameter fibers	Larger diameter fibers
Intracortical connections	Intrahemisheric and interhemisheric connections
Late myelinating	Early myelinating compared to SWM
Less myelin wraps around the axon	More myelin wraps around the axon
Complex arrangement	Less complex arrangement compared to SWM

Previous studies on HD have not focused on the superficial white matter, for example, although voxel (Di Paola et al., 2012), tract-based atlasing (Novak et al., 2014), and tractography methods (Phillips O. et al., 2013; Phillips et al., 2014, 2015) routinely find deep white matter impairments in Pre-HD and HD subjects, most analysis procedures are limited with regard to the spatial alignment of the cortical boundary, which is highly variable across subjects (Thompson et al., 2001; Smith et al., 2006). Furthermore, a recent study demonstrated that the complex arrangement of white matter fibers residing just under the cortical sheet (the superficial white matter) poses severe challenges for tractography (Reveley et al., 2015). Therefore, voxel- or tract-based atlasing methods lack the sensitivity for quantifying corticocortical connectivity at the juncture of the gray and white matter.

To address whether degeneration of the superficial white matter is associated with HD pathophysiology, we set out to assess if there was a difference in diffusion Magnetic Resonance Imaging (MRI) metrics between groups and then subsequently to assess the severity of changes. We predicted that diffusivity measures in the superficial white matter would be abnormal in Pre-HD subjects and these abnormalities would be worse in HD patients.

To achieve these goals, we investigated a sample of 98 subjects (49 healthy controls, 25 Pre-HD subjects, and 24 HD patients) by applying an advanced computational analysis approach that combines information from both diffusion and structural MRI data to allow local sampling of superficial white matter integrity measures. This approach, which is highly sensitive for extracting and comparing diffusion tensor imaging (DTI) metrics within the superficial white matter at the juncture of the gray and white matter (Phillips et al., 2011, 2016; Phillips O. R. et al., 2013) was applied to estimate and compare the effects of HD for axial, radial, mean diffusivity and fractional anisotropy (FA), at tens of thousands of spatially matched locations within the superficial white matter.

## Methods

### Subjects

Subject demographics and clinical assessments are outlined in Table 2. HD patients (*n* = 24) and Pre-HD subjects (*n* = 25), were examined clinically by neurologists with expertise in HD. All individuals were assessed using the Unified HD Rating Scale (UHDRS), which includes motor, cognitive, behavioral, and functional subscales (Kieburtz et al., 1996). Each section consists of a multistep subscale. The motor section measures eye movements, limb coordination, tongue impersistence and movement disorders (such as rigidity, bradykinesia, dystonia, chorea, and gait disturbances). A higher score means more motor impairment. The cognitive scale mainly evaluates executive function. A higher score means better cognitive performance. The behavioral section investigates the presence of depression, aggressiveness, obsessions/compulsions, delusions/hallucinations and apathy. A higher score means more impairment. The functional assessments include the HD functional capacity scale (HDFCS), the independence scale and a checklist of common daily tasks. All three scales mainly investigate independence in daily life activities. The HDFCS is reported as the total functional capacity (TFC) score (range 0–13) and is the only functional subscale with established psychometric properties (including inter-rater reliability and validity), which are based on radiographic measures of disease progression. Thus, the TFC score is used worldwide to determine patients' HD stage. On the independence scale, the investigator indicates whether the patient can perform the task that evaluates independence level (range 10–100). The checklist (functional assessment) is summed by giving a score of 1 to all “yes” answers (range 0–25). Pre-HD are defined as those subjects whom the suspected clinical diagnosis is confirmed by DNA analysis, which revealed (CAG)(n) expansion into the range characteristic of Huntington disease (> 36 or repeats), but who had a cognitive and behavioral assessment within the normality. We included in the Pre-HD group seven cases showing a motor score above 5, the chorea score (that is the most visible symptom) was very low and not exceeding 7 (a score below seven indicates that they did not have a score above 1 in each of the symptoms of the subscale). The Disease Burden index, a measure of disease severity, was used according to the already described formula (age × [CAG-35.5]), where CAG is the number of CAG repeats in *HTT* gene (Penney et al., 1997). A higher score reflects increased disease severity. The Mini Mental State Examination (MMSE) (Folstein et al., 1975), which measures global cognitive functioning, was administered to Pre-HD subjects and HD patients. A lower score reflects greater impairment.

**Table 2:** **Sociodemographic and clinical characteristics of patients and control subjects**.

	**Pre-HD (*n* = 25)**	**HD (*n* = 24)**	**Controls (*n* = 49)**	**Fisher's Exact Test; *F*-or T-Test**	***df***	***p***
**CHARACTERISTICS**						
Gender male/female	16/9	13/11	29/20	0.39,−0.40,−0.68	72, 71, 47	0.69^w^, 0.69^y^, 0.49^x^
Age (years ± SD)	37.44 ± 7.01	47.47 ± 14.76	42.41 ± 12.15	1.88,−1.68, −3.13	72, 71, 47	0.06^w^, 0.11^y^, 0.004^x^^a^
CAG repetition length	43.28 ± 2.17	46.67 ± 6.95	NA	−2.32	47	0.025^a^^††^
MMSE	27.82 ± 1.24	25.04 ± 3.30	NA	3.51	37	0.001^b^^††^
UHDRS Motor	6.48 ± 6.62	36.77 ± 13.30	NA	−8.44	43	0.001^a^^††^
UHDRS Cognitive	257.80 ± 42.34	143.50 ± 51.36	NA	7.82	40	0.001 ^b^^††^
UHDRS Behavioral	7.67 ± 7.84	18.41 ± 9.34	NA	−4.07	41	0.001^a^^††^
UHDRS Functional	25 ± 0	18.05 ± 5.77	NA	5.77	43	0.001^b^^††^
TFC	13 ± 0	8.41 ± 2.23	NA	9.09	43	0.001^b^^††^
Independence scale	99.8 ± 1.04	78.18 ± 12.77	NA	8.09	43	0.001^b^^††^
Disease Burden	292.3 ± 87.52	458.63 ± 106.97	NA	−5.50	47	0.001^a^^††^

*Mean Values are reported with the standard deviation. HD, HD; Pre-HD, gene-positive, without motor symptoms; SD, Standard Deviation; df, degrees of freedom; CAG, trinucleotide repeat number; MMSE, Mini-Mental State Examination; UHDRS, Unified HD Rating Scale; TFC, Total Functional Capacity; NA, not available*;

†† *Student's t-test, Bonferroni correction*.

w *= Pre-HD vs. Controls*.

y *= HD vs. Controls*.

x *= Pre-HD vs. HD*.

a *Pre-HD < HD (when it refers to age, a cognitive scale comparison or CAG repetition; higher punctuations mean greater impairment)*.

b *Pre-HD > HD (when it refers to a cognitive scale comparison; higher punctuations mean lesser impairment)*.

*MMSE: Missing data for 5 Pre-HD subjects & 5 HD patients; UHDRS Motor: Missing data for 2 Pre-HD subjects & 2 HD patients; UHDRS Cognitive: Missing data for 5 Pre-HD subjects & 2 HD patients; UHDRS Behavioral: Missing data for 4 Pre-HD subjects & 2 HD patients; UHDRS Functional: Missing data for 2 Pre-HD subjects & 2 HD patients; TFC: Missing data for 2 Pre-HD subjects & 2 HD patients*.

Fifty individually healthy subjects were recruited from the community (forty-nine had usable structural and diffusivity data). Patients in the advanced stages of disease (Stages III and IV) and/or with traumatic brain injury or focal lesions were excluded.

### Ethics statement

All participants had the cognitive capacity to understand the research protocol and gave their informed oral and informed written consent. Cognitive capacity to consent was determined by the MMSE. All subjects had previously performed a genetic test and were known to carry the CAG repeat expanded mutation (abnormal CAG repeats ≥36), in *HTT* gene. No subjects had severe cognitive impairment (see score on Table 2). In no case was a surrogate consent procedure carried out on the behalf of participants. Consent was obtained according to the Declaration of Helsinki and the Santa Lucia Foundation Research Ethics Committee approved the study.

### MRI data acquisition

All MRI data were acquired on a 3T Allegra MRI system (Siemens, Germany) using a birdcage head coil. Scans were collected in a single session, with the following pulse sequences: (1) T1-weighted 3D images, with partitions acquired in the sagittal plane using a modified driven equilibrium Fourier transform (Deichmann et al., 2004) sequence (TE/TR/TI: 2.4/7.92/910 ms, flip angle: 15°, 1 mm^3^ isotropic voxels); and (2) diffusion-weighted volumes were also acquired using SE echo-planar imaging (TE/TR: 89/8500 ms, bandwidth: 2126 Hz/voxel, matrix: 128 × 128, 80 axial slices, voxel size: 1.8 × 1.8 × 1.8 mm^3^) with 30 non-collinear distributed orientations for the diffusion sensitizing gradients at a *b*-value of 1000 s/mm^2^ and 6 *b* = 0 images. Diffusion-weighted scanning was repeated three times to increase the signal-to-noise ratio.

Images were also visually inspected for movement artifacts; subjects with excessive movement in their scans were excluded.

### Structural and diffusion MRI processing (Figure 1)

Detailed processing steps are covered in Phillips et al. (2016) with earlier iterations of the methods covered in Phillips et al. (2011) and Phillips O. R. et al. (2013). In brief, T1-weighted images were processed using BrainSuite's cortical surface extraction pipeline (http://brainsuite.org/processing/surfaceextraction/, V14B), which produces surface models of the cerebral cortex from T1 MRI (Joshi et al., 2007). Next, the surfaces for each subject were registered to a reference atlas surface using BrainSuite's surface/volume registration software (SVReg; http://brainsuite.org/processing/svreg/, V14B) (Joshi et al., 2004, 2007, 2012). This results in a spatial alignment of the white/gray matter boundary cortical surfaces across all subjects. Outputs from SVReg were inspected to ensure proper segmentation and surface/volume registration.

**Figure 1:**
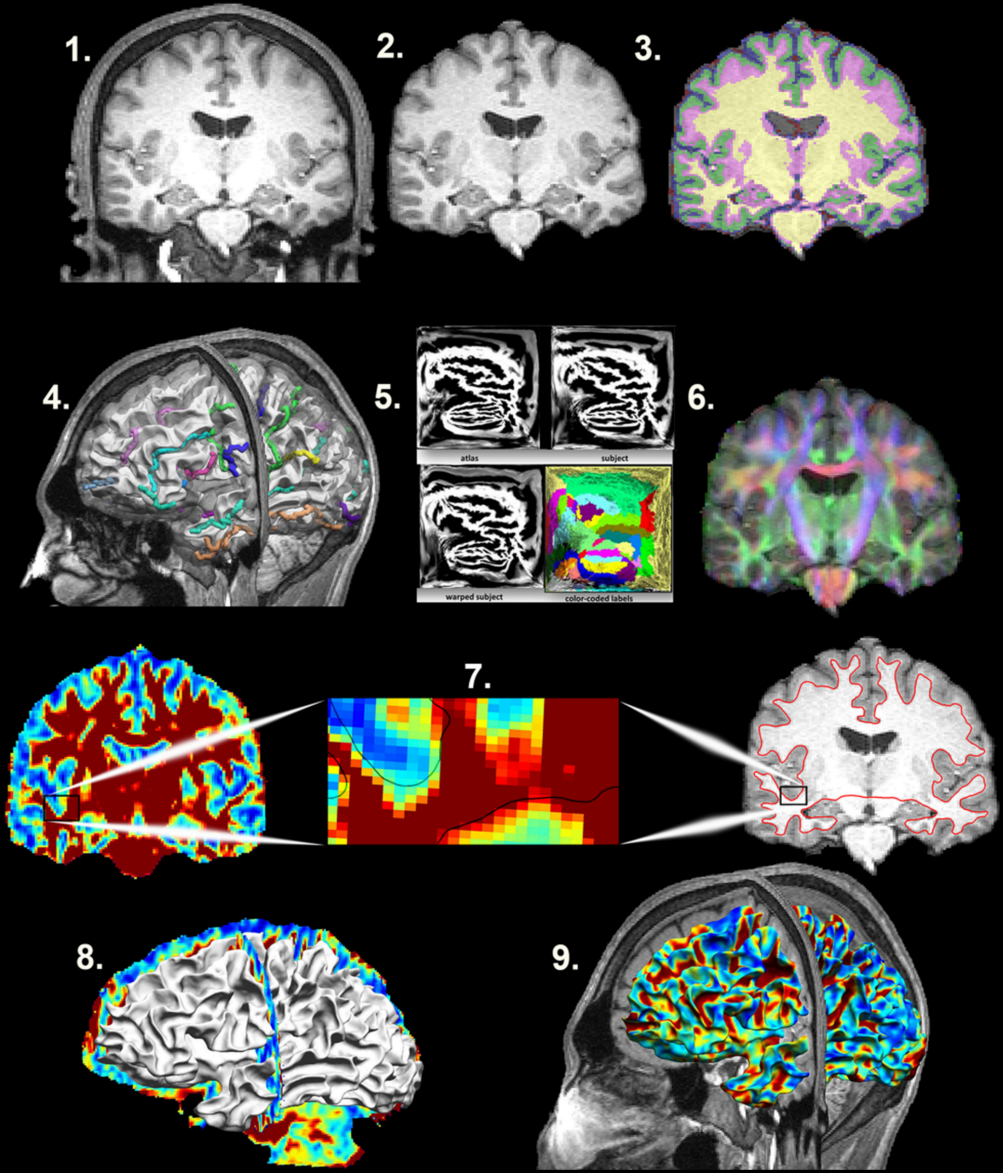
**Mapping the Superficial White Matter**. Processing steps in order to map the superficial white matter. 1. Original T1 structural image. 2. Bias field correction and skull stripping. 3. Tissue classification. 4. Surface extraction and sulcal line drawing. 5. Spatial alignment to the atlas. 6. Distortion correction and co-registration of the diffusion image to the structural MRI image. 7. FA and T1 images with zoom to show the surface boundary. 8. Superficial white matter spatially aligned surface and the FA image allows sampling of diffusivity values at each vertex of the surface. 9. Superficial white matter spatially aligned surface with FA estimated at each vertex. FA, Fractional Anisotropy.

Diffusion-weighted images were first processed with FMRIB's Software Library (FSL 4.1 www.fmrib.ox.ac.uk/fsl/). The output images where then processed with the BrainSuite Diffusion Pipeline (BDP Bhushan et al., 2015; http://brainsuite.org/processing/diffusion/). This included registration-based distortion correction using a constrained non-rigid registration based on mutual-information. BDP was used to fit tensor models to the diffusion MRI data, from which diffusion measures (FA, mean diffusivity, radial diffusivity, and axial diffusivity) were computed. Individual registrations were inspected visually to ensure proper alignment between the structural and diffusion images.

After T1 and DTI processing, a subcortical and ventricle mask was applied to the DTI images. Then the DTI images were smoothed using a 2-mm kernel. Finally, to allow cross-subject sampling of anatomically comparable superficial white matter axial/radial/mean diffusivity and FA, diffusivity images were sampled along each vertex of the white matter surface (158,748 vertices) using “Image To Shape Attributes” from the UCLA Shape Tools (Joshi et al., 2010, 2012) http://www.bmap.ucla.edu/portfolio/software/ShapeTools/.

### Statistical analysis

Demographic differences were assessed using chi-square, independent sample *t*-tests or with the General Linear Model as appropriate. Statistical analyses were performed using SPSS 20.0.

Superficial white matter axial/radial/mean diffusivity and FA values were averaged at each vertex point across the white matter surface separately for Pre-HD subjects, HD patients, and controls. The whole brain mean value was then extracted. These mean values were then analyzed using SPSS's General Linear Model with sex and age as covariates.

DTI measures with high spatial resolution across the superficial white matter surface were analyzed with the General Linear Model (http://brainsuite.org/bss/) (Joshi et al., 2014) to test for the effects of disease. Sex and age were included in the model as covariates. Surface based *p*-values were corrected using false discovery rate (FDR) correction (*p* = 0.05).

To calculate how much the DTI measures with high spatial resolution across the superficial white matter were different along each vertex between groups, percentage difference maps were created from the mean surface maps. These calculated the percentage difference at each vertex that Pre-HD subjects deviated from the control group, as well as HD patients from controls and finally Pre-HD subjects from HD patients for axial, radial, mean diffusivity, and fractional anisotropy.

Finally, to investigate whether superficial white matter changes were related to genotype, correlation analyses between CAG repeat length, and disease burden and whole brain superficial white matter diffusivity were performed. Sex and age were included as covariates, with the exception of Disease burden where age is already built into the measure.

## Results

*Subject demographics* are reported in Table 2. Pre-HD subjects and HD patients differed in age and CAG repetition length, but not in gender. Additionally, as expected, HD patients had significantly poorer performances with respect to all measures assessed by the UHDRS, and also a significantly higher score of Disease Burden.

### Whole brain superficial white matter findings (Figures 2, **3**)

**Figure 2:**
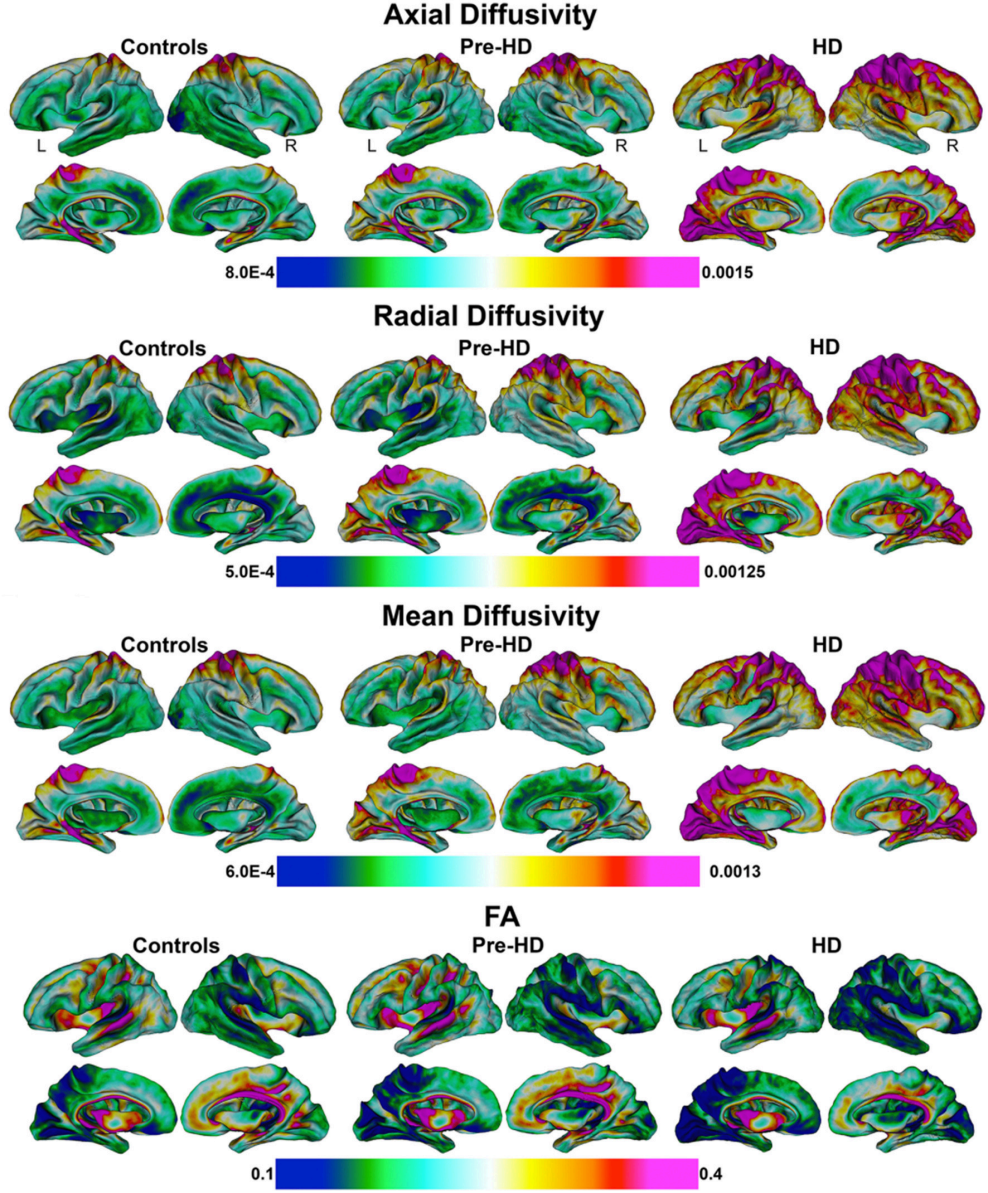
**Mean values of diffusivity parameters in Superficial White Matter**. Mean values of diffusivity parameters at each vertex in superficial white matter surfaces for each group (Controls, Pre-HD, and HD) (Axial/Radial/Mean diffusivity units: 10^−3^ mm^2^∕s). FA, Fractional Anisotropy.

Mean values of diffusivity measures at each vertex along the superficial white matter surface are shown in Figure 2.

**Figure 3:**
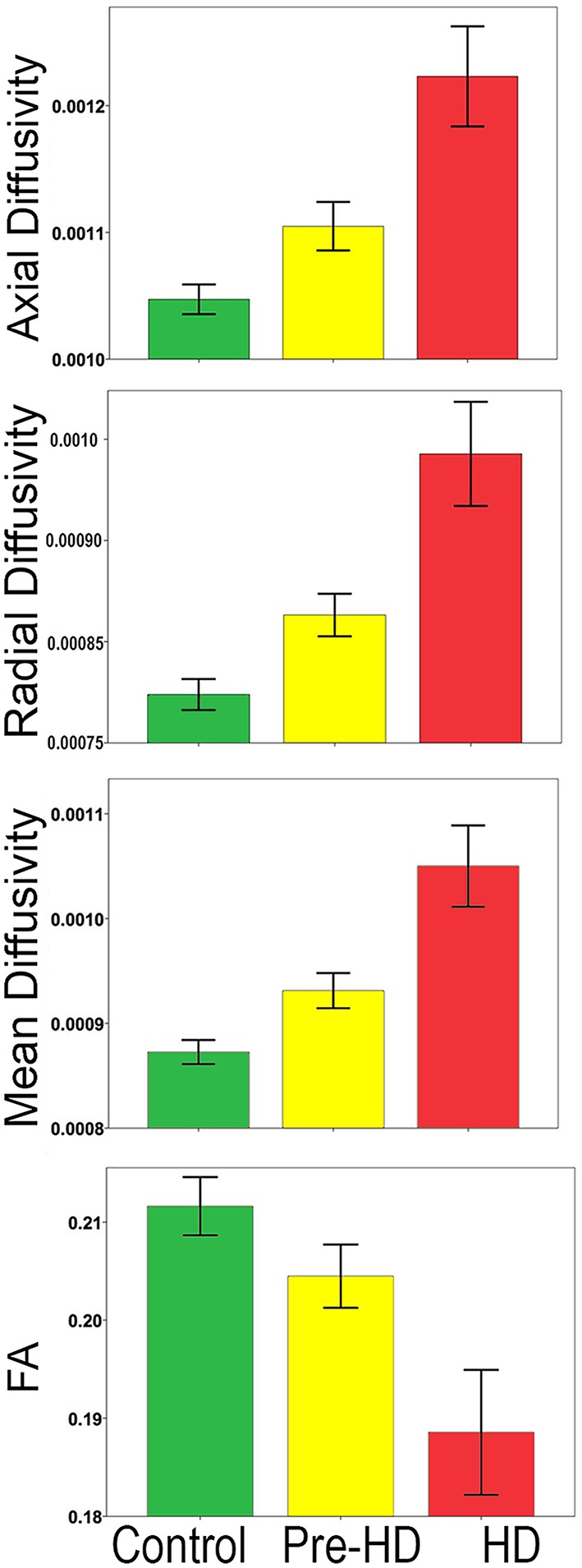
**Whole brain mean superficial white matter and group comparison**. Bar graphs show significant differences between Superficial White Matter AD, RD, MD, and FA. The error bars represent the Standard Error Mean (SEM). FA, Fractional Anisotropy; AD, Axial Diffusivity; RD, Radial Diffusivity; MD, Mean Diffusivity (AD/RD/MD Mean units: 10^−3^ mm^2^∕s).

Bar graphs in Figure 3 show the whole brain mean diffusivity measures for each group's superficial white matter. All diffusivity measures were significantly different between Pre-HD subjects and controls, and Pre-HD subjects and HD patients as well as between HD patients and controls. Pre-HD & HD Patients had increased axial, radial, and mean diffusivity and reduced FA compared to controls. Statistical details are outlined in Table 3.

**Table 3:** **Total superficial white matter group comparisons**.

**Region**		**Pre-HD**	**HD**	**Controls**	**MANOVA**			**Bonferroni *post-hoc* comparison (*p*)**		
								**Controls vs. Pre-HD**	**Controls vs. HD**	**Pre-HD vs. HD**
					***F***	***df***	***P***	***P***	***P***	***P***
	AD	1.11E-3 ± 4.60E-5	1.22E-3 ± 9.30E-5	1.047E-3 ± 4.63E-5	71.75	2.97	**0.001**	**0.001**	**0.001**	**0.001**
Total Superficial White Matter	RD	8.42E-4 ± 3.82E-5	9.64E-4 ± 9.14E-5	7.856E-4 ± 4.01E-5	80.04	2.97	**0.001**	**0.001**	**0.001**	**0.001**
(Mean value ±*SD*)	MD	9.31E-4 ± 4.08E-5	1.05E-3 ± 9.18E-5	8.738E-4 ± 3.98E-5	77.71	2.97	**0.001**	**0.001**	**0.001**	**0.001**
	FA	0.2045 ± 0.0078	0.1885 ± 0.0151	0.2116 ± 0.0103	34.43	2.97	**0.001**	**0.032**	**0.001**	**0.001**

*FA, Fractional Anisotropy; AD, Axial Diffusivity; RD, Radial Diffusivity; MD, Mean Diffusivity (AD/RD/MD Mean units: 10^−3^ mm^2^/s)*.

*Significant Bonferroni corrected results are in **BOLD***.

#### Disease effect of Pre-HD and HD on SWM: high resolution vertex based superficial white matter findings (Figure 4)

**Figure 4:**
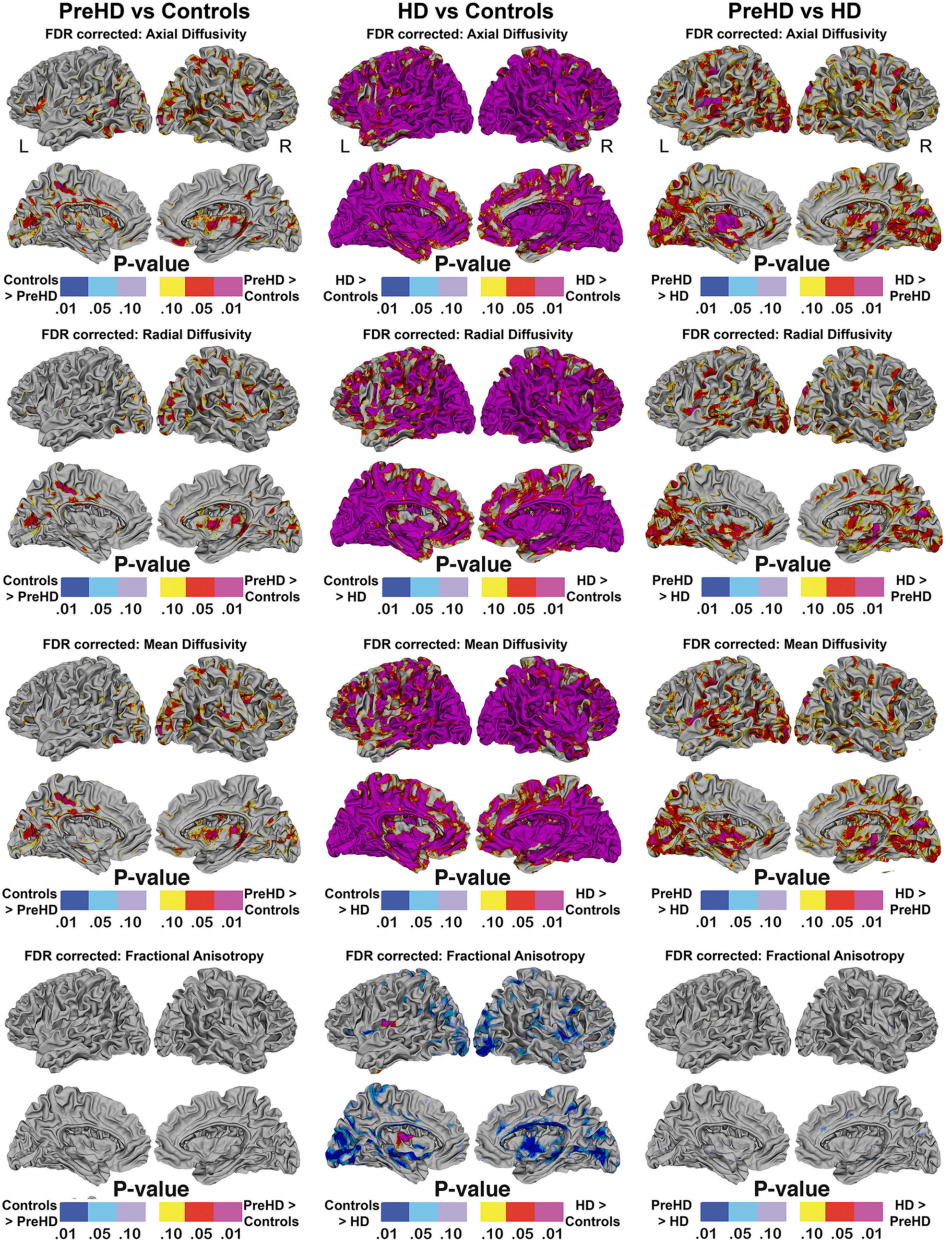
**Vertex based superficial white matter findings: Axial, Radial, Mean Diffusivity, and Fractional Anisotropy**. Probability maps showing FDR corrected effects of HD on the superficial white matter Axial, Radial, Mean Diffusivity, and Fractional Anisotropy controlling for age and gender mapped at high-spatial resolution at thousands of homologous locations within the superficial white matter. The direction of effects are indicated by the color bar. Red indicates increased diffusivity with disease and blue indicates reduced diffusivity with disease. FDR, false discovery rate.

Vertex based FDR-corrected statistical maps show the effects of HD for superficial white matter axial diffusivity, radial diffusivity, mean diffusivity, and FA at high spatial density corrected for age and gender (Figure 4). Only effects that survived FDR correction (FDR threshold = 0.05) are shown.

##### Axial/radial/mean diffusivity

The axial, radial, and mean diffusivity maps showed very similar data. However axial maps were slightly more sensitive to disease processes between Pre-HD and control subjects. Significant disease related increases were observed between Pre-HD subjects and controls in a disperse manner across much of the brain. The posterior portion in both hemispheres was particularly affected. When comparing HD patients vs. controls subjects, significant changes were observed in all parts of the brain with only a few areas appearing to be spared such as the left anterior frontal pole, and the bilateral anterior cingulate (though with a major degree in axial diffusivity), and the anterior mesial portion of the temporal pole.

Pre-HD subjects vs. HD patients displayed significant changes in most parts of the brain that were prominent in the occipital lobe bilaterally as well as the left motor area.

##### Fractional anisotropy

**Figure 5:**
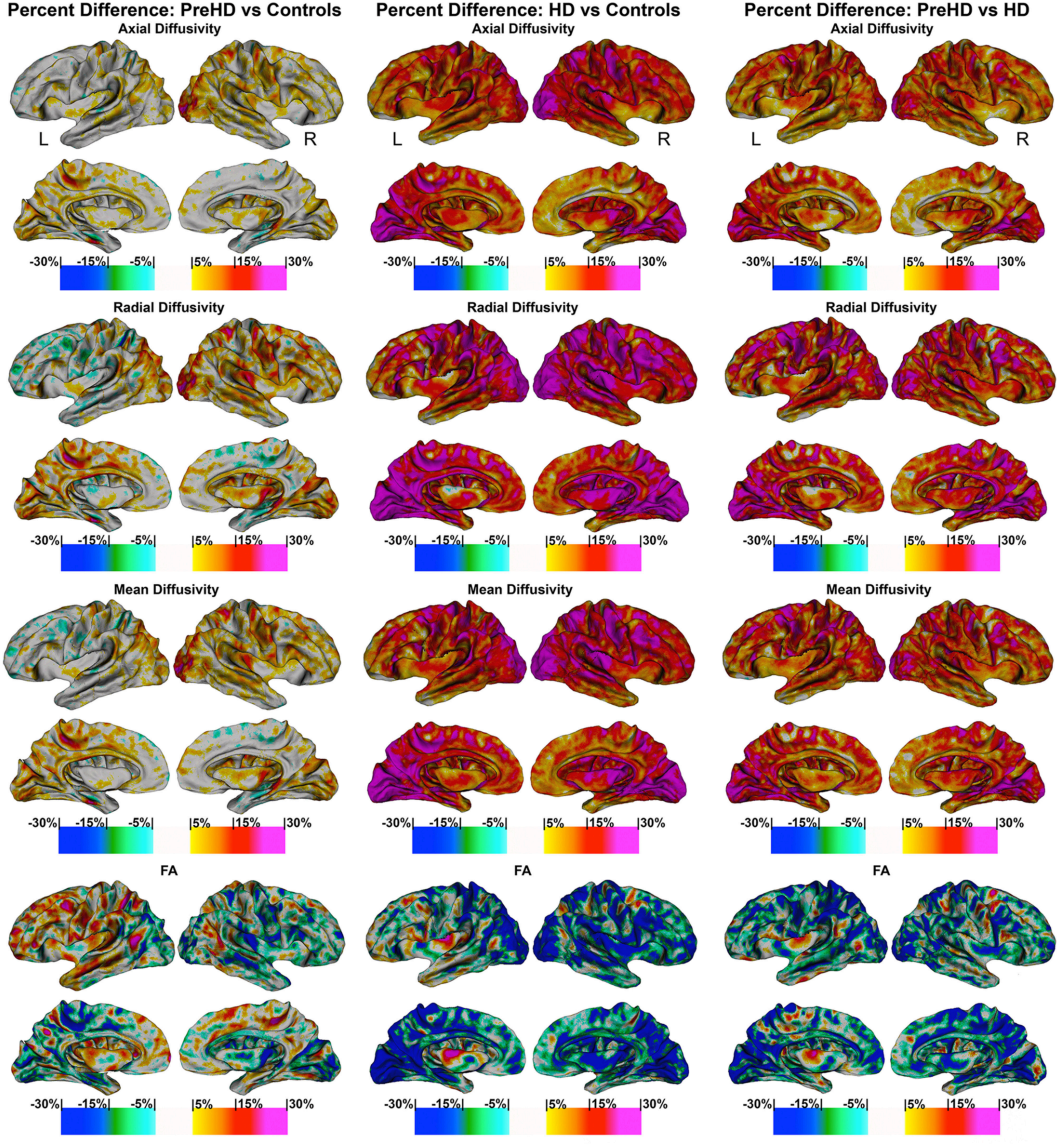
**Percentage change maps: axial, radial, mean diffusivity and fractional anisotropy**. Vertex based percentage change maps show the difference between Controls and Pre-HD subjects, Controls and HD patients, and Pre-HD subjects and HD patients superficial white matter axial diffusivity, radial diffusivity, mean diffusivity, and fractional anisotropy at high spatial density. For axial, radial, and mean diffusivity, red colors represent higher diffusivity in Pre-HD subjects and HD and blue colors represents lower diffusivity when compared to controls. For HD patients vs. Pre-HD red colors indicate increased diffusivity for HD patients. For fractional anisotropy, blue colors represent decreased fractional anisotropy for Pre-HD and HD patients compared to controls. For HD patients vs. Pre-HD, blue colors indicate lower fractional anisotropy for HD patients.

Pre-HD subjects did not show reduced fractional anisotropy compared to controls that survived FDR correction. However, HD patients had reduced fractional anisotropy compared to controls in the occipital lobe as well as the temporal lobe and right frontal lobe. HD patients had increased fractional anisotropy in a small portion along the right insula (not visible in view shown in Figures 4, 5).

#### Disease effect of Pre-HD and HD on SWM: high resolution vertex based superficial white matter percentage change between groups (Figure 5)

The maps show the brain changes in percentages for superficial white matter axial diffusivity, radial diffusivity, mean diffusivity, and fractional anisotropy at high spatial density (Figure 5).

##### Axial diffusivity

When comparing Controls and Pre-HD subjects, Pre-HD subjects had higher diffusivity across much of the brain that ranged from 5 to 15% where the right hemisphere had more widespread increases on the lateral surface and the left hemisphere had more widespread increases on the medial surface. In the left hemisphere increased diffusivity was largely confined to the occipital and parietal lobe as well as the superior temporal with large increases in diffusivity up to 20% in the paracentral lobule. In the right hemisphere, diffusivity increases reached greater than 25% in the occipital lobe and ranged from 5 to 20% in sensorimotor regions. Compared to the rest of the brain the frontal lobe was relatively spared.

When comparing HD patients to controls, diffusivity increases that ranged between 5 and 30% covered almost all the brain. In the bilateral occipital lobes, increases of >25% covered much of the area as well as the parietal and the frontal lobes (motor area). Only the temporal pole and the anterior portion of frontal lobe had patches that were spared from diffusivity increases.

When comparing Pre-HD subjects and HD patients, HD patients had large increases in diffusivity between 5 and 30% over all of the brain in a widespread manner that were greatest in the occipital and parietal and frontal lobes (motor area). Again temporal and frontal pole appeared relatively spared.

##### Radial diffusivity

Results were very similar to axial diffusivity, however, for both comparisons between Pre-HD subjects and controls and HD patients and controls, there were greater increases in the Pre-HD and HD groups for diffusivity compared to the axial diffusivity maps. The left frontal lobe (rostral to motor area) had lower diffusivity in Pre-HD subjects compared to controls (5–10%).

When comparing HD patients to controls, and comparing Pre-HD subjects and HD patients, we found results similar to those reported for the axial diffusivity map.

##### Mean diffusivity

This showed an intermediate rate of change between the axial and radial percentage change maps. When comparing Pre-HD and controls subjects there were large increases of 10–25% across much bilateral occipital lobes and between 5 and 20% increases across much of the right hemisphere. HD patients had large increases in diffusivity (10–30%) across much of the brain compared to controls and Pre-HD compared to HD maps showed similar but with lower increases in diffusivity.

##### Fractional anisotropy

FA was more varied compared to axial and radial maps. Pre-HD subjects had lower fractional anisotropy (5–15%) in the occipital lobes compared to controls and across much of the right hemisphere. The left frontal lobe had higher fractional anisotropy in Pre-HD subjects compared to controls that ranged between 5 and 15%. HD patients had large decreases (10–20%) in the occipital lobe and across the parietal lobe when compared to controls as well as more spare decreases in the temporal and frontal lobes. Pre-HD compared to HD maps were similar where HD patients had decreased FA across much of the brain but to a lesser degree when HD patients were compared to control subjects.

#### Correlations with CAG repeat and disease burden (Figure 6)

When correlating CAG repeat length to the mean superficial white matter diffusivity it was significantly positively correlated with axial diffusivity (*r* = 0.424, *P* < 0.003), radial diffusivity (*r* = 0.435, *P* < 0.002), mean diffusivity (*r* = 0.432, *P* < 0.002) and significantly negatively correlated with fractional anisotropy (*r* = −0.355, *P* < 0.014). Disease burden was also significantly positively correlated with axial diffusivity (*r* = 0.456, *P* < 0.001), radial diffusivity (*r* = 0.459, *P* < 0.001), and mean diffusivity (*r* = 0.459 *P* < 0.001), and negatively with fractional anisotropy (*r* = −0.380, *P* < 0.007).

**Figure 6:**
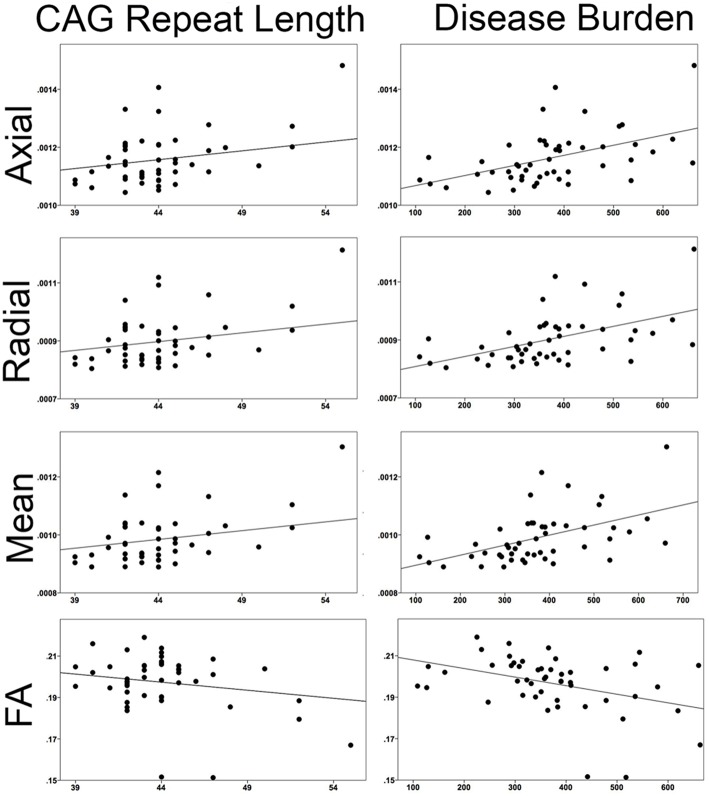
**Correlations between superficial white matter and measures of disease severity**. Mean superficial white matter diffusivity measures correlated with CAG repeat length and Disease Burden. All correlations were significant. (Axial/Radial/Mean diffusivity units: 10^−3^ mm^2^∕s).

## Discussion

This study sought to identify whether or not the superficial white matter was abnormal in Pre-HD and HD patients. The following main findings emerged from the investigation: (1) there are broad abnormalities in superficial white matter of Pre-HD subjects that are greatest in the occipital lobe and motor areas; (2) HD patients show strikingly pervasive damage in all parts of the superficial white matter; (3) the superficial white matter changes are strongly correlated with the HD genotype (CAG repeat length, disease burden).

There are a growing number of studies on the deep white matter in HD. These (our own on the same subjects presented here and others using different subjects) point to abnormal white matter in Pre-HD patients with damaged white matter in HD patients (Reading et al., 2005; Rosas et al., 2006, 2010; Dumas et al., 2012; Di Paola et al., 2014; Matsui et al., 2014; Phillips et al., 2014, 2015). Our findings on superficial white matter changes are in line with this large body of work on the deep white matter. However, the superficial white matter differs from the deep white matter in several ways. For example, it is a dense complex layer of interconnected fibers (Reveley et al., 2015), it is late myelinating and particularly sensitive to the effects of age (Bartzokis, 2004; Phillips O. R. et al., 2013) and in early myelinating deep white matter, oligodendrocytes tend to myelinate a single axon segment with over 100 myelin membrane wraps, while late-myelinating regions (of which the superficial white matter is part of) contain oligodendrocytes which may myelinate as many as 50 axon segments with fewer than 10 wraps (Butt and Berry, 2000). This makes the late myelinating oligodendroctyes structurally more complex and metabolically overextended (Peters and Sethares, 2004; Haroutunian et al., 2014). While the present study is not directly studying these cells, they are found in high concentrations in the superficial white matter. As such, they could play an important role in the large differences in diffusivity found between groups. However, this aspect has not been directly studied. Furthermore, the biological process underlying variation in diffusivity is complex (Jones et al., 2012), and interpreting axial and radial values is difficult (Wheeler-Kingshott and Cercignani, 2009). Decreased FA indicates the loss of water directionality likely due to damage of the structural organization of the tissue. It's worth highlighting that axial, radial and mean diffusivity were much more sensitive to disease processes than FA. This finding is in line with a number of other DTI studies on HD (Dumas et al., 2012; Phillips et al., 2014, 2015). Furthermore, because of the complex arrangement of fibers in the superficial white matter, the difference between axial and radial diffusivity values is smaller than in the deep white matter, this suppresses the usefulness of FA as a measure in this region. Regardless of the complications in interpreting diffusivity measures in the superficial white matter, the abnormalities presented can be seen as a manifestation of the pathologies' evolution.

The abnormalities in the superficial white matter can clearly be seen in an examination of the average diffusivity taken from across the white matter surface (Figure 3). This finding shows a large difference in diffusivity between Pre-HD subjects and controls and demonstrates that the superficial white matter is already abnormal before symptoms are present. To examine the specificity of these differences, we used a precise vertex-wise approach, which gave both the statistical significance at each point along the superficial white matter as well as how much each group varied from one another (percentage difference). When comparing HD patients and controls, a quick look at the P-maps between HD patients and controls (Figure 4) demonstrates that HD patients have statistically significant damage that covers essentially the whole brain. This is a major finding and it demonstrates just how widespread damage to the superficial white matter is in HD patients. However, because the damage is so widespread, in order to elucidate information about a possible disease progression we need to look at the percentage change maps (Figure 5). Here we can see that the greatest increases in diffusivity (25–30%) are largely located in the occipital, parietal and motor regions, while the anterior portions of the temporal and frontal lobes had comparatively lower increases in diffusivity (10–20%). To gain further insight, we can examine the contrast between controls and Pre-HD subjects. Here, although the changes are much more subtle, the statistically significant changes are still widespread. However, the greatest increases in diffusivity were seen in the occipital lobes and in and around the motor regions (greater than a 20% increase in diffusivity compared to controls). This is interesting because although the superficial white matter is the last region to myelinate, the pattern of myelination across the superficial white matter likely follows the normal pattern of brain development where the motor and visual areas develop early compared to language and executive areas (temporal and frontal; Phillips O. R. et al., 2013). Thus our finding of pre-symptomatic disease related changes in early maturing areas compared to late maturing regions suggests that the superficial white matter may develop normally in people with a high number of CAG repeats, but as the superficial white matter reaches myelination maturation they become vulnerable to disease processes.

To help us understand how the superficial white matter is affected by the pathological mechanisms underlying HD, we can examine the contrast between Pre-HD and HD groups. When comparing Pre-HD subjects to HD patients, both the left and right occipital lobes and motor areas had increased diffusivity in HD patients. Since these regions were already damaged in Pre-HD subjects, it indicates that these regions become more damaged as the disease progresses. This is also the case for the rest of the brain such as the parietal lobe, which had large increases between 5 and 30% as well as the areas in and around the sensory and motor regions (Figure 5). Furthermore, the temporal and frontal lobes also had rapid deterioration that ranged from 5 to 20%, however, this was less severe in the most anterior portions of these lobes. These findings appear to indicate a posterior to anterior disease progression. This is clearly an oversimplification of the disease progression and more work will be needed to elucidate the precise progression using a longitudinal sample. However, the general pattern does appear to fit the posterior to anterior progression that has been shown to take place in the corpus callosum (Phillips O. et al., 2013; Di Paola et al., 2014), in the deep white matter (Phillips et al., 2014), and along the gray matter of the cortex (Rosas et al., 2008). Since the brain matures in a general posterior to anterior direction, this lends support to the hypothesis that the brain develops largely normally in HD patients, but as it matures mutant HTT becomes detrimental to the brain.

Finally, the disease effect maps (Figure 4) are similar to the changes that have been shown to take place in the cortical gray matter of the cortex (Rosas et al., 2008; Phillips et al., 2015), however, here they almost encompass the whole brain. This indicates that damage to the superficial white matter likely occurs before or separately from damage to the cortical gray matter–or this approach is more sensitive to disease processes.

Mapping the superficial white matter using diffusivity values is very sensitive to disease processes; however, some potential study limitations are worth discussing. Future studies including higher angular resolution DTI data might help confirm the regional specificity of superficial white matter disconnectivity in HD and clarify relationships with specific functional impairments. Also, the superficial white matter surface is generated from the T1 image, which is not optimized to classify white and gray matter. Future studies could benefit from high-field MRI, which could possibly help improve delineation at the juncture of the gray and white matter. Finally, a recent study demonstrated that partial volume effects near the corpus callosum could alter diffusion MR indices in Huntington's disease (Steventon et al., 2016). In order to minimize potential volume effects in this area, we masked out the ventricles.

## Conclusion

There is evidence that the deep white matter is damaged in Pre-HD subjects (Phillips et al., 2014, 2015). Now we have revealed widespread damage to the superficial white matter even before symptoms are present. This indicates that superficial white matter is very sensitive to HD pathological processes. Damage advances in a posterior to anterior direction with early myelinating regions being most susceptible. Given the accumulating evidence, it is becoming increasingly clear that that abnormal white matter is one of the major components of HD pathophysiology.

## Author contributions

OP participated on methods, study design, data collection, and analysis as well as writing the manuscript. SJ, DS, KN all helped with methods development. Manuscript preparation was done with the help of SJ, KN, FS, CS, CC, US, MD. Clinical advice was given by MD, FS, CS, CC, and US. Study design was done by MD and OP and analysis methods were created with SJ. Data collection was done in conjunction with CS and US.

### Conflict of interest statement

The authors declare that the research was conducted in the absence of any commercial or financial relationships that could be construed as a potential conflict of interest.

## Abbreviations

HD: Huntington's disease
PreHD: Pre-symptomatic Huntington's disease
DTI: diffusion tensor imaging
FA: fractional anisotropy
MRI: magnetic resonance imaging.
